# Hybrid fixation in the bilateral sagittal split osteotomy for lower jaw advancement

**DOI:** 10.1590/S1678-77572010000100015

**Published:** 2010

**Authors:** Felipe Ladeira PEREIRA, Marcos JANSON, Eduardo SANT'ANA

**Affiliations:** 1 DDS, Oral and Maxillofacial Surgeon, Brazilian Army, Juiz de Fora General Hospital (HGeJF), Juiz de Fora, MG, Brazil.; 2 DDS, MS. Orthodontist, Private Practice, Bauru, SP, Brazil.; 3 DDS, PhD, Associate Professor of Oral and Maxillofacial Surgery, Bauru School of Dentistry, University of São Paulo, Bauru, SP, Brazil.

**Keywords:** Bilateral sagittal split osteotomy, Orthognathic surgery, Monocortical fixation, Bicortical fixation, Mandibular advancement, Condylar torque

## Abstract

Miniplate and screw fixation has been widely used in bilateral sagittal split osteotomy, but some issues remain unclear concerning its lack of rigidity when compared to Spiessl's bicortical technique. This paper demonstrates the hybrid fixation technique in a case report. A 34-year-old female patient underwent a double jaw surgery with counter-clockwise rotation of the mandible fixed using the hybrid fixation technique. The patient evolved well in the postoperative period and is still under follow up after 14 months, reporting satisfaction with the results and no significant deviation from the treatment plan up to now. No damage to tooth roots was done, maxillomandibular range of motion was within normality and regression of the inferior alveolar nerve paresthesia was observed bilaterally. The hybrid mandibular fixation is clearly visible in the panoramic and cephalometric control radiographs. It seems that the hybrid fixation can sum the advantages of both monocortical and bicortical techniques in lower jaw advancement, increasing fixation stability without significant damage to the mandibular articulation and the inferior alveolar nerve. A statistical investigation seems necessary to prove its efficacy.

## INTRODUCTION

Bilateral sagittal split osteotomy (BSSO) is commonly used to treat mandibular discrepancies^7,11,28^. The ability to rigidly and properly fix the fractured segments at the time of surgery may facilitate healing in the immediate postoperative period and reduce the displacement possibility of the bony segments, particularly the condylar proximal segment^27^. The technique that uses bicortical compressive screws was first described by Spiessl^25^ (1974) while the technique that uses miniplates and monocortical screws was introduced by Luhr^14^ (1986).

Monocortical osteosynthesis has been widely used in the fixation of BSSO^8^, leading to stable results according to the literature^3,8,15,19-21^, in spite of being considered as a semi-rigid fixation^21^. Since monocortical fixation is not as rigid as bicortical osteosynthesis, the excessive shear force stress, produced by the compressive action of the masseter muscle to the osteotomy line, may transform the mandibular shape postoperatively^9,18^. On the other hand, other authors have found no differences in the stability promoted by both techniques^8,11,26^.

Concerning the surgical treatment in Class II patients, fixation should be stable and precise enough to allow great advance without compromising the bone healing and stability. In skeletal Class II malocclusion, the following characteristics can be observed, alone or in association: mandibular retrusion; vertical deficiency or excess of the maxilla and maxillomandibular retrusion^17^.

This paper describes, through a case report, the routine use of the hybrid fixation technique for BSSO in mandibular advancement, which associates the advantages of two commonly used techniques: the positional bicortical screws and the monocortical plate osteosynthesis.

## CASE REPORT

A 34-year-old female patient searched Dr. E.S. complaining the "lack of chin", "reversed lower lip" and gummy smile. The patient had previous orthodontic treatment with dental compensation, having both maxillary first premolars already extracted and the gap closed. Clinically, the patient presented symmetric dolicocephalic face with maxillomandibular retrusion; maxillary vertical excess; chin deficiency and an accentuated facial convexity. In addition, healthy periodontal tissues and temporomandibular joints (TMJs), tension of the orbicularis oris and mental muscles during function and lower lip incompetency when relaxed were also observed.

**Figure 1:**
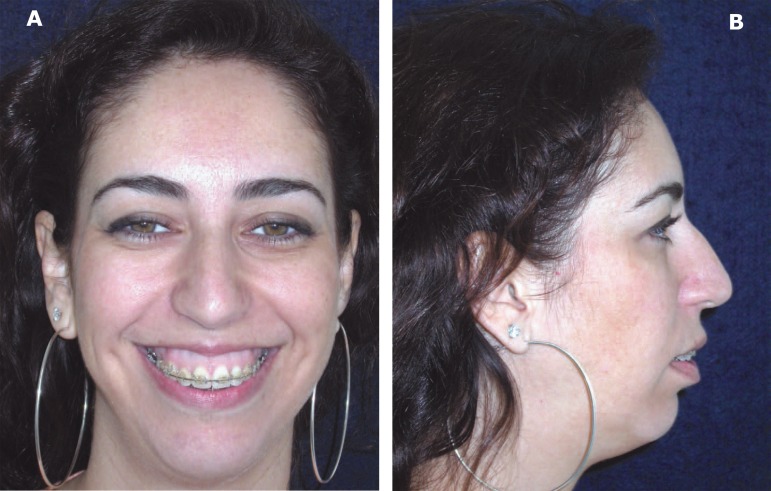
Preoperative frontal aspect (A) and facial profile (B) of the patient (patient signed informed consent authorizing the publication of these pictures)

**Figure 2:**
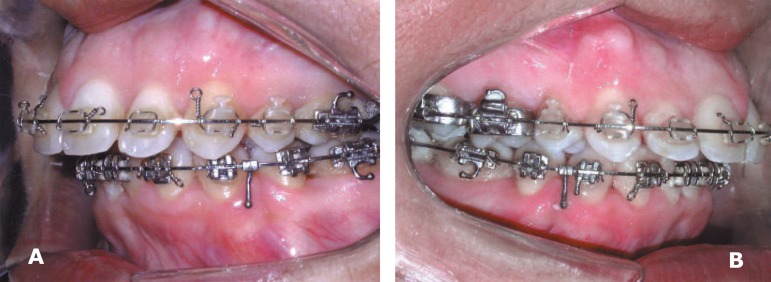
Preoperative intraoral view of the left side (A) and right side (B)

In order to acquire proper positioning of the mandibular incisors, the right and left first premolars were extracted and levelling and alignment of the maxillary arch was performed to create a positive overjet (Figures 1 and 2). For the planning, Arnett, et al.^2^ (1999) soft tissue analysis was used. The surgical plan consisted in maxillary impaction and advancement, mandibular counter-clockwise rotation with an overall advancement of 9 mm in B point, 13.7 mm in Pog and 3 mm of genioplasty. Under general anesthesia, the mandible was managed and fixed as described below, followed by usual maxillary Le Fort I.

**Figure 3:**
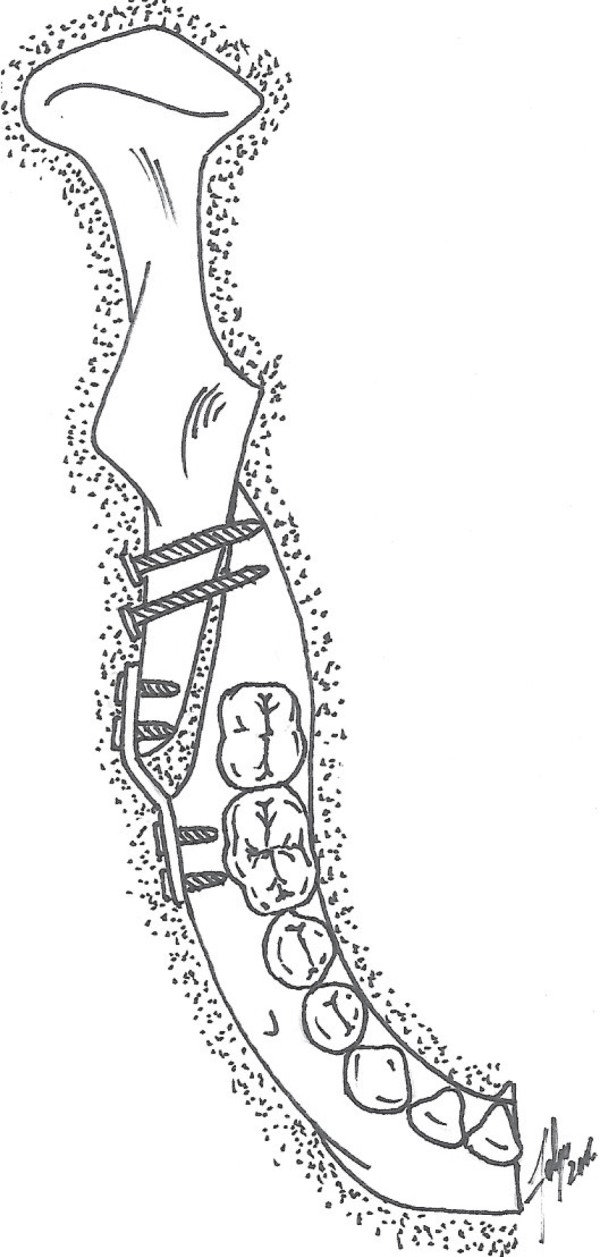
Schematic illustration of the hybrid fixation in the BSSO. Note the inset bend at the plate

After BSSO according to Epker's^6^ (1977) modified technique, the condyle was properly positioned^1^ and the osteotomy fixed with a 2.0 mm miniplate and two monocortical screws in each segment. An inset bend was made at the plate to maintain the gap and avoid condylar torque^1,19,20^. Having the 4 screws in position, a transorally oblique perforation was drilled in the retromolar region (visualising the proximal end of the distal fragment), the hole was tapped and a 2.0 mm diameter x 16 mm long screw was inserted. A second screw was placed distally from the first in the same manner. These screws were positional and do not exert pressure between segments (Figure 3). The same fixation was done on the other side. After completion of the osteosynthesis, maxillomandibular immobilization (MMI) was removed and occlusion and mouth opening were checked. The wounds were then sutured as usual.

**Figure 4:**
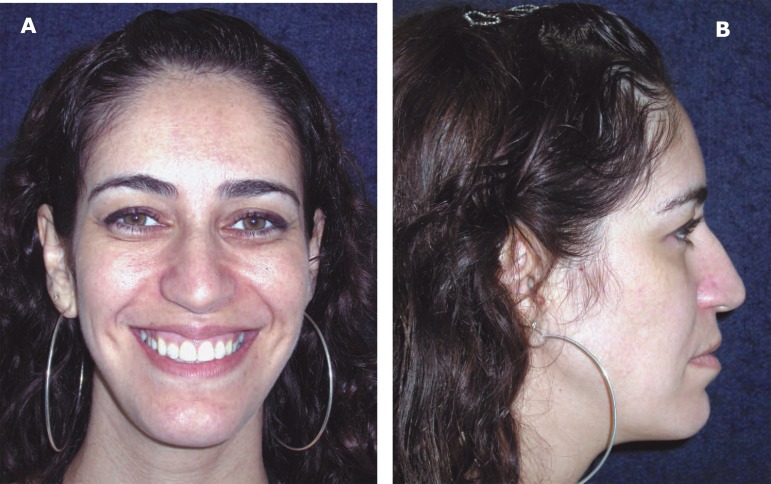
Postoperative frontal aspect (A) and facial profile (B) of the patient (patient signed informed consent authorizing the publication of these pictures)

**Figure 5:**
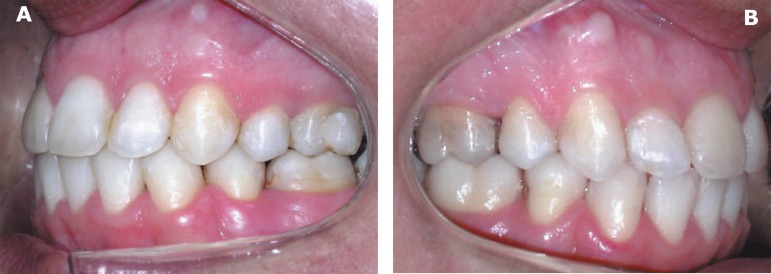
Postoperative intraoral view of the left side (A) and right side (B)

**Figure 6:**
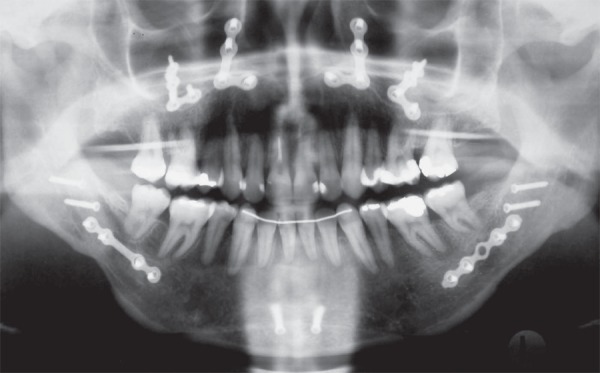
Panorex showing hybrid fixation in mandibular advancement

**Figure 7:**
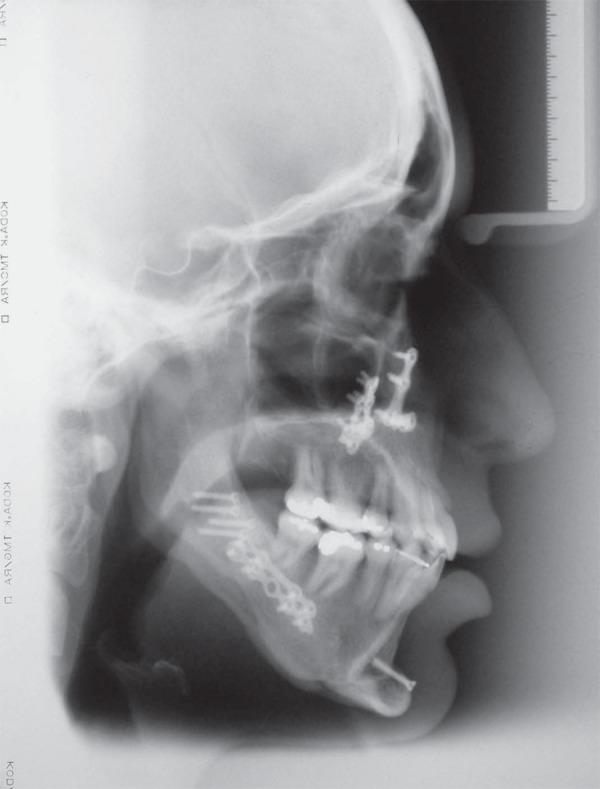
Fourteen-month cephalometric radiograph showing occlusion plane changing and lower jaw advancement

**Figure 8:**
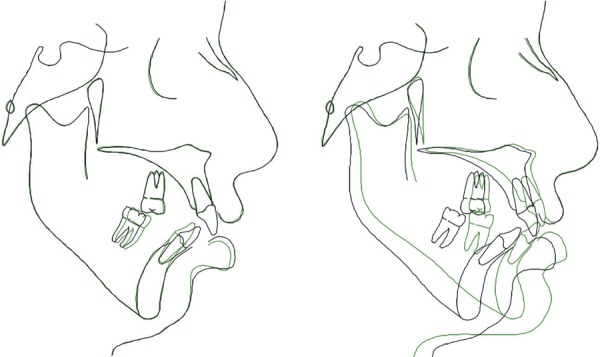
Cephalograms taken 1 week before (left) and 1 week after (right) surgery showing pre-surgical orthodontic treatment and the counter-clockwise rotation of the lower and upper jaws after surgery

The patient evolved well in the postoperative period and is still under follow up after 14 months, reporting satisfaction with the results (Figures 4 to 5). No damage to tooth roots was done, maxillomandibular range of motion is within normality and regression of the inferior alveolar nerve (IAN) paresthesia is observed bilaterally. The hybrid mandibular fixation is clearly visible in the panoramic and cephalometric control radiographs (Figure 6 and 7). Figure 8 shows cephalometric tracing of the preoperative position and postoperative changes along the 16 months of treatment, including the removal of brackets.

## DISCUSSION

The hybrid fixation technique in the BSSO with one 2.0 mm miniplate, four monocortical and two positional screws was initially described for management of cases where the lingual cortical plate of the distal segment had fractured^27,28^. The purpose of the suggested technique is to be the routine fixation method in mandibular advancements in order to increase stability of the single 4-hole miniplate while maintaining its advantages, such as: lower possibility of IAN compression, absence of skin scars (since the retromolar screws are placed transorally) and passive condyle accommodation at the glenoid fossa. Moreover, the earlier release of the elastic MMI in the postoperative period, the sooner the patient will start soft diet ingestion (while using guiding elastics for intercuspation) without significant increase of the total fixture cost.

Among the drawbacks of the bicortical screw technique are IAN compression, scars in the face or neck made by the transcutaneous perforation and rotation of the mandibular condyles^1,3,9,11,19,20,23^. In the method proposed for this case, mandibular third molar extraction prior to surgery is necessary for bicortical screw placement. Since extractions are not absolutely necessary when miniplates are the only fixation method, it can be considered an advantage of this technique^18^.

Concerning the miniplate advantages, the three-dimensional relationship between the segments is established by the miniplate, with the condyle in the glenoid fossa and the proximal and distal segments in their initial contact point^21^. No compression is made between the segments and the result is immediate and functionally stable^3,8,15,19-21^. The miniplate applied in the anterior border of the buccal osteotomy facilitates manipulation of the proximal segment and seating of the condyle; after fixed it is stable enough to permit release of the MMI and intraoperative inspection of the occlusion^23^. Any corrections may be easily achieved at this stage by releasing and reattaching the distal ends of the miniplates^19,23^. Also, plates can be easily removed transorally in the postoperative period if necessity rises^19^. Moreover, passive plate bending and application helps maintaining the axial condylar orientation within the fossa^19^. It is also conceivable that the fragments fixed by miniplates, which are bent to accommodate the step at the buccal surface, would tend to cause less harm to the IAN^20,23^, and also reduces the risk of damaging the roots^11^.

After monocortical osteosynthesis have been applied, small forces directed across the osteotomy can still change the relative positions of the segments. This can lead to occlusal changes when patients return to function early or when they are noncompliant^21^. The unpredictable fixation provided by the miniplates alone may compromise the clinical outcome if the patient is restored to early function^23^. Therefore, if the patient's postsurgical occlusion is unstable, monocortical osteosynthesis will lead to too much rotation of the mandible and may cause delayed union and breakage of the miniplates^4,8^. Therefore, especially in these patients or those who underwent an overcorrection, osteosynthesis should be performed bicortically^8^.

The fixation relapse rate in the bicortical screws technique ranges from 8 to 11%^12,13^, while for the miniplates this value ranges from 5.2 to 15%^3,19,20^. According to a recent literature review^10^, bicortical screws show only slight differences regarding skeletal stability compared to miniplates in short-term, but a large number of studies with higher skeletal long-term relapse rates were seen in patients treated with bicortical screws instead of miniplates. By advancing the mandible, the submandibular soft tissue drape is stretched together with the suprahyoid and infrahyoid muscles. As a consequence, the hyoid, fixed by these muscles, is pulled forward, but will return to its original position several months postoperatively. Stretching of theses tissues gives rise to a constant force opposite to the vector of the mandibular advancement^1,3,11,16^. Relapse seems to be a multifactorial phenomenon affected by many variables, such as (from strongest to weakest evidence): amount of advancement; type and material of fixation; low and high mandibular plane angle; control of proximal segment; soft tissue and muscles; remaining growth and remodeling; preoperative age and surgeon skills^10^. Although relapse can occur after 6 months, the greatest amount of relapse occurs in the early postoperative time (6 weeks to 6 months)^5,16,29^. A correlation between the amount of advancement and relapse may occur only when the advancement exceeds 7 mm^3,10,16,19,29^. Furthermore, the MMI period in the first weeks seems to reduce the relapse rate^5,16^, without great significant risks of muscular atrophy if this time is short^16^.

In a study investigating the skeletal stability following sagittal split osteotomy using monocortical miniplate internal fixation, Rubens, et al.^19^ (1988) observed that all patients who presented some sort of symptom related to the TMJ in the preoperative period had its resolution in the postoperative period, while three patients (15%) that were symptom-free before surgery, started having symptoms after surgery. In a similar study, Scheerlinck, et al.^20^ (1994) noted that among patients who presented TMJ dysfunction in the preoperative period, 68% showed improvement or resolution, 20% noted no difference and 12% reported worsening of the symptoms. Among the patients who didn't present symptoms of TMJ dysfunction in the preoperative period, 80% still had no complaints in the postoperative period, 13% had muscular pain, 5.5% an intermediate click and 1.5% closed lock^20^. Kahnberg, et al.^11^ (2007) reported that more than a half of the pre-surgical symptomatic patients had its signs and symptoms solved in the postoperative period, while approximately 25% of those without TMJ dysfunction in the preoperative period developed it after surgery. Nevertheless, in a study investigating sagittal split advancement osteotomies stabilized with miniplates, approximately 7% of the patients underwent progressive condylar resorption (PCR), which generated relapse in the B-point, and whose initial signs are usually seen 6 months postoperatively, showing a direct connection between the amount of advancement and the risk of PCR^20^. In fact, the endorotation movement that happens in the TMJ in mandibular advancements has major potential to cause dysfunction in the TMJ than the exorotation observed in surgeries for mandibular prognathism^19^.

Shetty, et al.^24^ (1994), in a study using a biomechanical model of the BSSO, showed that, within a physiologic range of loading, their hybrid technique using one positional screw (different from the method reported in this paper), produced stability that was comparable with or superior to that produced by conventional methods of rigid internal fixation. Because the plate and the monocortical screws are placed first, a bone clamp is never applied across the osteotomy^21^. The segment clamping negates advantage of the positional screw, and makes it function as a lag screw^21^. This could produce lingual or rotational movement of the proximal segment, with consequent condylar displacement, occlusal changes and TMJ problems, in addition to the possibility of IAN compression between the segments, producing numbness or paresthesia^1,12,13,21,23^. Since the positional bicortical screw from the technique is placed far anteriorly than those normally placed in the Spiessl's^25^ (1974) technique, it can be placed transorally, avoiding a transcutaneous stab incision and thus an unfavorable scar^21,22^. In the technique described for the present case, the two non-compressive bicortical screws are easily placed transorally, by angulating the drill and inserting the screw while visualizing if the gap between the fragments stays still. If the gap starts to increase, the screw should be removed, reangulated and reinserted. When the gap is greater than 2 mm, particulated bone graft are usually used.

The screw applied bicortically in the retromolar region inhibits the displacement tendencies through its resistance to axial and shear forces^9,23^. A second screw, as proposed in our technique, would guarantee the immobility of the segments. The use of 2 miniplates on each side to increase fixation stability in great mandibular advancements has been described^7,15,23^. In the technique proposed here, stability can be increased without the expense of these additional two plates and eight screws usually necessary in these cases, but using four bicortical screws, two on each side, lowering the total fixture costs. Within the anatomic limits imposed by the BSSO, it is known that the greater the separation between the retromolar screw and the miniplate, the better the expected functional stability^23^.

The goal of the technique proposed in this paper is to associate the rigidity of the bicortical positional screws with the advantages of the monocortical miniplates in lower jaw advancement, without increasing the treatment cost or TMJ damage, allowing early release of the MMI and probably reducing the relapse rate. Further investigations comparing clinical relapse rate of the suggested technique to other technique are still necessary.
